# Engineering a Plant-Derived Astaxanthin Synthetic Pathway Into *Nicotiana benthamiana*

**DOI:** 10.3389/fpls.2021.831785

**Published:** 2022-01-18

**Authors:** Quinton M. Allen, Vicente J. Febres, Bala Rathinasabapathi, José X. Chaparro

**Affiliations:** ^1^Horticultural Sciences Department, University of Florida, Gainesville, FL, United States; ^2^Plant Molecular and Cellular Biology Program, Horticultural Sciences Department, University of Florida, Gainesville, FL, United States

**Keywords:** astaxanthin, *Nicotiana benthamiana*, *Adonis aestivalis*, *Brevundimonas* sp. SD212, Agrobacterium-mediated transformation

## Abstract

Carotenoids have been shown to be essential for human nutrition. Consumption of carotenoid-rich fruits and vegetables can reduce the risk of many diseases. The ketocarotenoid astaxanthin has become a commercially valuable compound due to its powerful antioxidant properties compared to other carotenoids. It is naturally produced in certain algae, bacteria, and the flowers of some species of the genus *Adonis*, although it is produced in such small quantities in these organisms that it is costly to extract. Chemical synthesis of this compound has also shown limited success with a high proportion of esterified forms of astaxanthin being produced, which decreases antioxidant properties by the conversion of hydroxyl groups to esters. Previously, transgenic astaxanthin-producing plants have been created using a β-carotene ketolase enzyme of either bacterial or algal origin. However, a novel astaxanthin pathway exists in the flowering plants of the genus *Adonis* which has not been utilized in the same manner. The pathway involves two unique enzymes, β-ring-4-dehydrogenase and 4-hydroxy-β-ring-4-dehydrogenase, which add the necessary hydroxyl and ketone groups to the rings of β-carotene. In the present study, *Nicotiana benthamiana* plants were transformed with chimeric constructs coding for these two enzymes. The regenerated, transgenic plants accumulate astaxanthin and their growth (height and weight) was unaffected, when compared to non-transformed *N. benthamiana* and to plants transformed with the bacterial β-carotene ketolase. The accumulation of astaxanthin also improved seedling survivability under harsh UV light, mitigated reactive oxygen accumulation, and provided a phenotype (color) that allowed the efficient identification and recovery of transgenic plants with and without selection.

## Introduction

**Figure 1:**
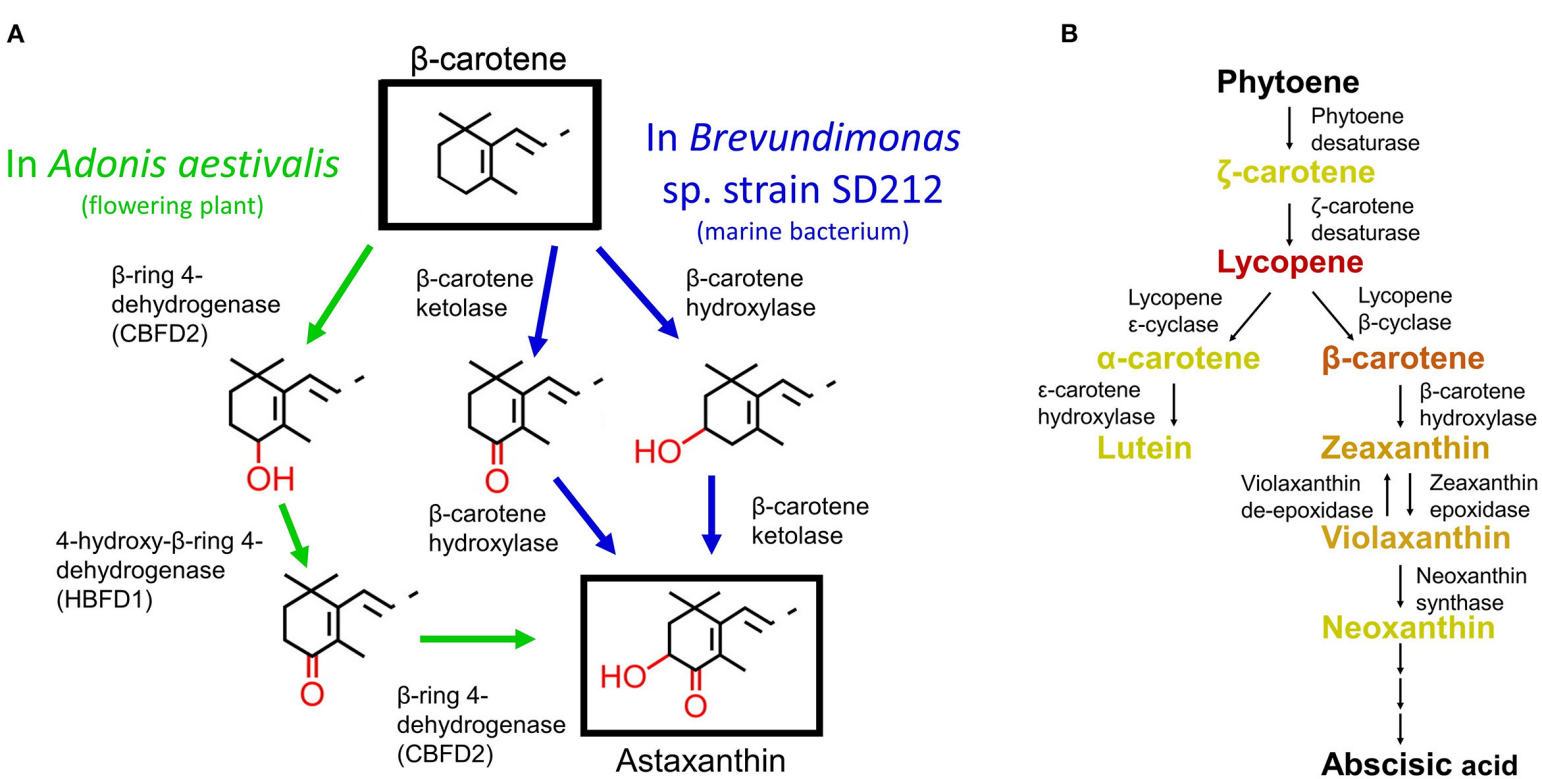
**(A)** Two routes of astaxanthin biosynthesis from β-carotene in nature. The pathway with green arrows details the three-step reaction involving the two enzymes in *Adonis aestivalis*. The pathway with blue arrows details the two-step reaction involving two enzymes from *Brevundimonas* sp. SD 212. The two separate blue pathways indicate that the two steps can occur in either order. **(B)** Simplified carotenoid pathway present in most higher plants. The names of the compounds are color coded to symbolize the actual color of the molecules. Names written in black are colorless in nature. Abscisic acid is not a full-length carotenoid, but rather an important apocarotenoid derivative. **(A)** modified from Cunningham and Gantt (2011) and **(B)** modified from Wei et al. (2014).

Carotenoids are an important class of dietary compounds due to their nutraceutical properties. They are found in most living organisms whether produced endogenously or acquired through diet and are responsible for most red, orange, and yellow pigments seen in nature. One carotenoid, astaxanthin, is a high-valued compound due to its increased antioxidant potential relative to other carotenoids and the limited number of organisms that produce it naturally (O'Connor and O'Brien, 1998). It is also used industrially as a food colorant, dietary supplement, and in aquaculture as a feed supplement. Structurally, astaxanthin is a β-carotene molecule with hydroxyl groups added at the 3,3' and ketone groups at the 4,4' of the β-rings (Higuera-Ciapara et al., 2006) (Figure 1A). Due to these additional oxygen-containing groups, astaxanthin has higher antioxidant potential compared to other carotenoids more commonly synthesized in plants.

In the last few decades, the astaxanthin biosynthetic pathways in several species of bacteria and algae, a species of yeast, *Xanthophyllomyces dendrorhous*, and the *Adonis* genus of flowering plants have been elucidated (Johnson and An, 1991; Fraser et al., 1997; Visser et al., 2003; Cunningham and Gantt, 2011; Liu et al., 2020). Genes from the conserved pathway in algae and bacteria have been used to transgenically induce astaxanthin biosynthesis in plants that otherwise do not produce it naturally (Morris et al., 2006; Hasunuma et al., 2008; Huang et al., 2013; Harada et al., 2014; Pierce et al., 2015; Nogueira et al., 2017; Zhu et al., 2018). These experiments were aimed at creating efficient sources of astaxanthin for industrial extraction or producing plants with a value-added trait. The most common transgenic approach has been the addition of a β-carotene ketolase from either bacteria (*Brevundimonas* sp. SD212) or algae (*Haematococcus pluvialis*). In this approach, the β-carotene ketolase is responsible for the addition of the 4,4'-ketone groups on the β-carotene molecule (Figure 1A). Most higher plants possess an endogenous β-carotene hydroxylase as part of their core carotenoid pathway (Figure 1B). This enzyme adds the 3,3'-hydroxyl groups to β-carotene. A previous study found that expressing an exogenous β-carotene hydroxylase in addition to the β-carotene ketolase increased the accumulation of astaxanthin in transgenic plants (Hasunuma et al., 2008).

An alternative approach to using the β-carotene ketolase from algae or bacteria is to utilize the enzymes found in the genus *Adonis* (Figure 1A). This genus of flowering plants produces high levels of astaxanthin in its flower petals; however, the petals also produce toxic compounds preventing their use as a source for commercial extraction (Cunningham and Gantt, 2005). The enzymes responsible for the conversion of β-carotene into astaxanthin, β-ring 4-dehydrogenase (CBFD) and 4-hydroxy-β-ring 4-dehydrogenase (HBFD), were previously identified through *E. coli* complementation, however they have not been studied in other plant systems. These enzymes convert β-carotene into astaxanthin through a series of three reactions on each β-ring of β-carotene. First, a hydroxyl group is added to the 4 position on the aromatic ring by CBFD, then that hydroxyl group is converted into a carbonyl group by HBFD, then finally another hydroxyl group is added at the 3 position by CBFD (Cunningham and Gantt, 2011). These two enzymes use only β-carotene as a substrate, whereas the β-carotene ketolase of either bacterial or algal origin can use zeaxanthin, β-cryptoxanthin or β-carotene as substrates, leading to the production and potential accumulation of multiple intermediates. The sequential activity of the two *Adonis* enzymes coordinate the efficient production of astaxanthin from the substrate (Cunningham and Gantt, 2011), whereas many combinations of the β-carotene ketolase and β-carotene hydroxylase enzymes are not well-coordinated and are inefficient at producing astaxanthin (Morris et al., 2006; Zhong et al., 2011; Huang et al., 2013; Pierce et al., 2015). In addition, *Adonis* genes have optimal codon usage ideal for transgenic expression in flowering plants compared to algal or bacterial genes and they already possess an *N*-terminal transit peptide to target the protein product into the plastid.

The objective of this research was to evaluate CBFD and HBFD from *Adonis* in *Nicotiana benthamiana* to determine whether this was a feasible route to metabolically engineer astaxanthin production. Plants harboring the *Adonis* genes under constitutive promoters were compared to transformants with the bacterial β-carotene ketolase. To determine the effect the transgenes had on leaf carotenoid metabolism and plant development, carotenoid content and growth rate were measured on the regenerated transgenic plants. Additionally, due to the high antioxidant potential of astaxanthin, the plants were assayed for reactive oxygen species (ROS) accumulation and response to excess UV.

## Results

### *N. benthamiana* Transformation

**Figure 2:**
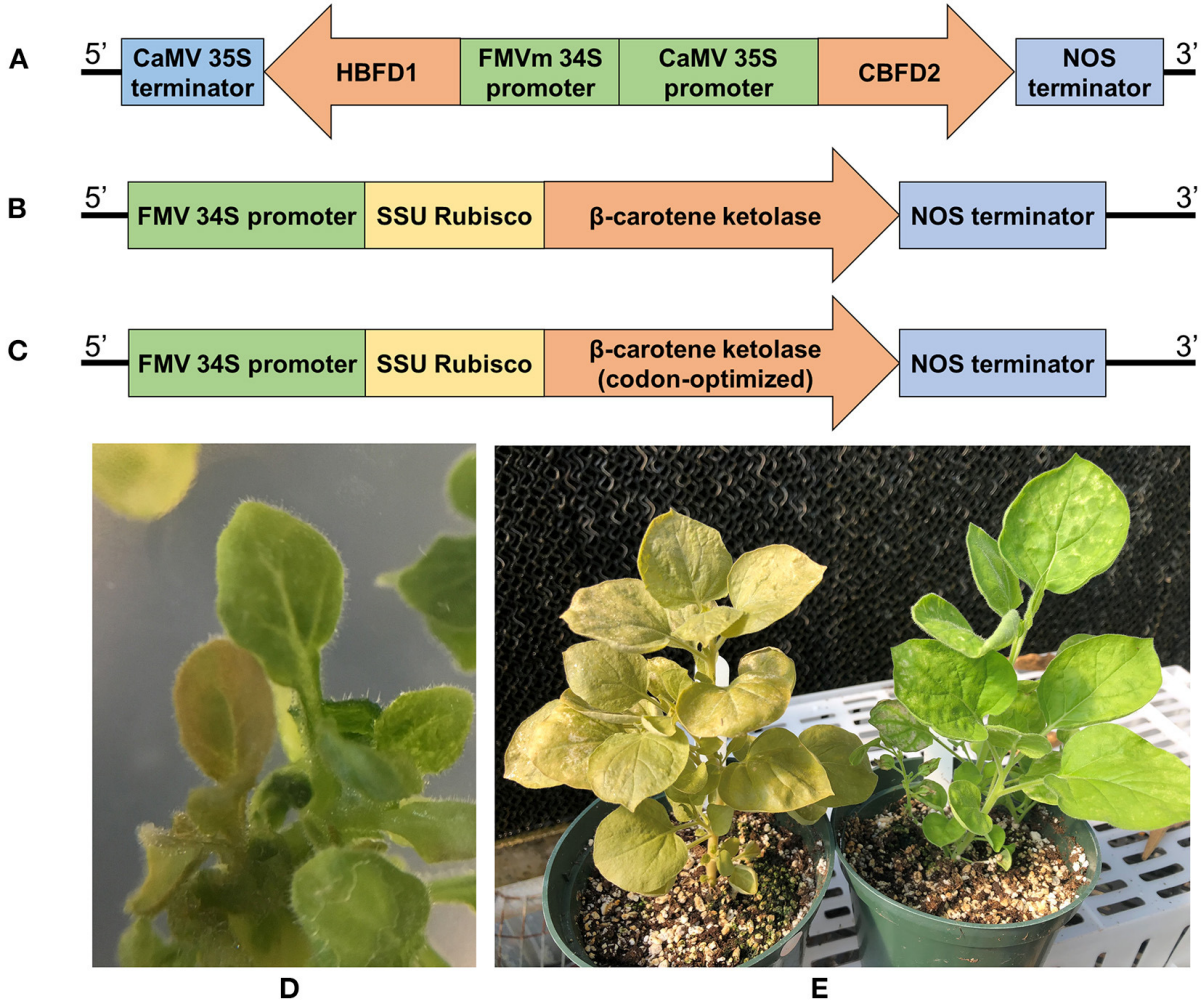
**(A–C)** Schematic representation of the gene constructs used to induce astaxanthin production in the transgenic plants. All constructs were cloned in pCAMBIA2201 which contains *GUS* and Kanamycin resistance genes for expression in plants and chloramphenicol resistance for bacterial selection (not shown). **(A)** pCAMBIA2201-HBFD1-CBFD2 plasmid contains the two astaxanthin inducing chimeric genes, *HBFD1* and *CBFD2*, from *Adonis aestivalis*, **(B)** pCAMBIA2201-crtW plasmid contains the β-carotene ketolase, **(C)** pCAMBIA2201-Cit/crtW plasmid contains the plant codon-optimized cDNA for β-carotene ketolase. FMVm 34S promoter, Figwort mosaic virus minimal 34S promoter; CaMV 35S T, Cauliflower mosaic virus 35S terminator; NOS, nopaline synthase terminator. **(D)** Transformed T0 *Nicotiana benthamiana* plant showing the characteristic copper color. **(E)** Color comparison between astaxanthin and non-astaxanthin accumulating plants 3 months after transformation. On the left is a pCAMBIA2201-HBFD1-CBFD2 transformant and on the right is a pCAMBIA2201 empty vector transformant. This copper-colored phonotype was consistent between all astaxanthin-accumulating plants regardless of the construct harbored.

Three constructs were designed to compare the most utilized astaxanthin pathway using *crtW* to the alternative route in *Adonis aestivalis*. One construct, pCAMBIA2201-HBFD1-CBFD2 (Figure 2A), had both *HBFD1* and *CBFD2* from *A. aestivalis* under the control of a bidirectional constitutive promoter, consisting of a full-length cauliflower mosaic virus (CaMV) 35S promoter and minimal figwort mosaic virus (FMV) 34S promoter. The other two constructs, pCAMBIA2201-crtW (Figure 2B) and pCAMBIA2201-Cit/crtW (Figure 2C), contained native and *Citrus* codon-optimized versions of the *crtW* gene from *Brevundimonas* sp. SD212 with full length FMV 34S promoters, respectively. Due to the bacterial origin of the *crtW* gene, a plant codon-optimized version of *crtW* was synthesized to evaluate any effects on translation efficiency due to the origin of the compared enzymes. The codons were optimized for *Citrus* for use in future research applications. A plastid transit peptide sequence was also fused to the *N*-terminus of the crtW and Cit/crtW proteins to ensure transport into the chloroplast, the location of carotenoid synthesis in plants. *N. benthamiana* was transformed and plants were successfully regenerated with all three constructs (Table 1). Transformants (confirmed by PCR) possessed a phenotype with an easily visible copper-color in all tissues due to the accumulation of astaxanthin (Figures 2D,E). The intensity of the copper-colored phenotype was consistent between plants with all three constructs.

**Table 1:** Transformation efficiency of *N. benthamiana* on media with antibiotic selection compared to media without antibiotic selection.

	**With kanamycin selection**			**Without kanamycin selection**		
**Construct**	**Total number of regenerants**	**GUS positive regenerants**	**Transformation efficiency^a^**	**Total number of regenerants**	**GUS positive regenerants**	**Transformation efficiency**
pCAMBIA2201-crtW	76	13	21%	163	8	5%
pCAMBIA2201-Cit/crtW	141	26	23%	158	10	7%
pCAMBIA2201-HBFD1-CBFD2	126	25	25%	171	13	8%
pCAMBIA2201	77	14	22%	134	5	4%

a *Transformation efficiency was calculated as the number of GUS positive regenerants divided by the total number of regenerants*.

### Carotenoid Analysis

**Figure 3:**
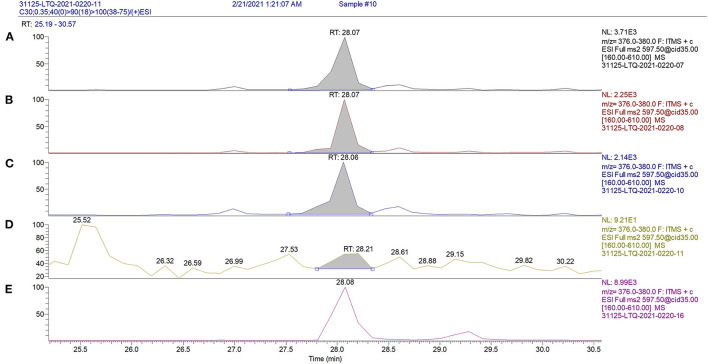
Identification of astaxanthin in T1 *N. benthamiana* plants through the mass peaks of product ions generated by HPLC/ESI(+)-MS/MS. The graph shows the 379.2 m/z mass peak from a plant expressing crtW **(A)**, Cit/crtW **(B)**, HBFD1 and CBFD2 **(C)**, empty vector **(D)** and an astaxanthin analytical standard **(E)**. HPLC/ESI(+)-MS/MS = high performance liquid chromatography, coupled with tandem mass spectrometry with positive electrospray ionization.

Total carotenoids were extracted from leaves of T1 *N. benthamiana* transformed plants to verify astaxanthin production and accumulation levels via high performance liquid chromatography coupled with positive electrospray ionization followed by tandem mass spectrometry (HPLC/(+)ESI-MS-MS). Because carotenoid biosynthesis is tightly coordinated, the levels of other important carotenoid intermediates including β-carotene, zeaxanthin and lutein levels were also measured. Additionally, canthaxanthin was measured, which is an intermediate in the astaxanthin pathway that is only produced in astaxanthin producing organisms. Structurally, canthaxanthin is a β-carotene molecule with 4,4'-ketone groups added and measuring this compound can provide data on the efficiency of astaxanthin synthesis from β-carotene. In all cases, the transformed vector control and wild type *N. benthamiana* plants had no detectable astaxanthin, with a minimum detection limit of 1 μg/g of dry tissue. For each construct, three independently transformed lines were analyzed with two technical replicates of each sample and astaxanthin was identified and the levels measured through mass spectral traces (Figure 3). Of the examined transgenic lines, the highest astaxanthin-accumulating lines were HBFD1-CBFD2 2-5 (0.99 mg/g) and 1–8 (0.56 mg/g), and Cit/crtW 2–4 (0.40) and the lowest were Cit/crtW 2-16 (0.13 mg/g), HBFD1-CBFD2 2-6 (0.15 mg/g), and crtW 1-1 (0.16 mg/g) (Table 2). Additionally, the percent of total carotenoids that consisted of astaxanthin ranged from 3.3% (Cit/crtW 2-16) to 26.5% (HBFD1-CBFD2 2-5) (Table 2). There was a wide range in the amount of astaxanthin produced between lines and constructs, however there were no significant differences in total carotenoids between any of the astaxanthin-accumulating lines (Supplementary Table S1).

**Table 2:** Quantification of the carotenoids astaxanthin, β-carotene, and canthaxanthin in T1 *N. benthamiana* leaves using high performance liquid chromatography coupled with mass spectrometry.

		**Astaxanthin**			**β-carotene**			**Canthaxanthin**		
**Construct**	**Line**	**mg/g of dry tissue**	**% of total carotenoids**	**SG^a^**	**mg/g of dry tissue**	**% of total carotenoids**	**SG**	**mg/g of dry tissue**	**% of total carotenoids**	**SG**
pCAMBIA2201-crtW	1-1	0.16 ± 0.03	5.4%	cd	0.38 ± 0.02	12.7%	c	0.006 ± 0.001	0.18%	b
pCAMBIA2201-crtW	1-10	0.24 ± 0.04	7.0%	bcd	0.49 ± 0.22	14.4%	bc	0.012 ± 0.001	0.31%	a
pCAMBIA2201-Cit/crtW	2-4	0.40 ± 0.10	11.8%	b	0.37 ± 0.06	10.9%	c	0.006 ± 0.001	0.16%	b
pCAMBIA2201-Cit/crtW	2-5	0.18 ± 0.05	5.0%	bcd	1.48 ± 0.09	41.3%	ab	0.003 ± 0.000	0.08%	bc
pCAMBIA2201-Cit/crtW	2-16	0.13 ± 0.04	3.3%	cd	1.23 ± 0.11	31.1%	abc	0.003 ± 0.002	0.06%	bc
pCAMBIA2201-HBFD1-CBFD2	1-8	0.56 ± 0.24	16.7%	b	0.99 ± 0.27	29.5%	abc	0.003 ± 0.000	0.08%	bc
pCAMBIA2201-HBFD1-CBFD2	2-5	0.99 ± 0.14	26.5%	a	0.99 ± 0.22	26.5%	abc	0.002 ± 0.000	0.06%	bc
pCAMBIA2201-HBFD1-CBFD2	2-6	0.15 ± 0.05	5.6%	cd	0.88 ± 0.44	32.6%	abc	0.001 ± 0.001	0.03%	c
pCAMBIA2201	1-5	0.00 ± 0.00	0.0%	d	1.39 ± 0.53	39.9%	abc	0.000 ± 0.000	0.00%	c
Non-transformed	–	0.00 ± 0.00	0.0%	d	1.66 ± 0.12	50.8%	a	0.000 ± 0.000	0.00%	c

a *SG, significance group*.

*Values are means ± standard error of four replicates. Significance groups between experimental lines were determined by Tukey's HSD using variance calculated with ANOVA. α = 0.05. Minimum detection limits for the compounds were 1 μg/g of dry tissue*.

The β-carotene content in the astaxanthin-accumulating lines ranged from 0.37 mg/g in the plants expressing crtW to 1.48 mg/g in the plants with Cit/crtW (Table 2). The non-transformed control plants had significantly more β-carotene than the crtW lines as well as Cit/crtW 2-4. There was no significant correlation (Pearson's correlation coefficient = −0.22) between astaxanthin level and β-carotene level (Data not shown).

Astaxanthin and β-carotene comprised most of the carotenoid content in these plants, however there are other nutritionally relevant carotenoids that could have been affected by astaxanthin biosynthesis. Zeaxanthin and lutein are important carotenoids for photosynthesis and canthaxanthin is an astaxanthin intermediate and thus were measured in addition to astaxanthin and β-carotene. These three compounds constituted <2.0% of the total carotenoids in all samples, including controls (Table 2; Supplementary Table S1). Zeaxanthin, which can also serve as a precursor to astaxanthin synthesis, accounted for 0.3% or less of the total carotenoids in all lines. Plants with HBFD1-CBFD2 had a range of zeaxanthin content from 2 to 6 μg/g of dry tissue (Supplementary Table S1). Zeaxanthin levels in lines possessing HBFD1-CBFD2 were not significantly different from the non-astaxanthin accumulating lines. Similarly, lutein levels in HBFD1-CBFD2 lines ranged from 20 to 38 μg/g of dry tissue, which only accounted for 0.5–1.1% of total carotenoids, respectively (Supplementary Table S1). However, there were no significant differences in the amounts of lutein in any of the lines measured. Canthaxanthin, which is a keto-carotenoid intermediate to astaxanthin not normally produced by *N. benthamiana*, was observed in amounts ranging from 0.03 to 0.31% of the total carotenoids in the transgenic lines (Table 2). Line crtW 1-10 had significantly more canthaxanthin than all other lines measured.

### Phenotype Evaluation

Growth rate over 8 weeks was measured to determine any phenotypic differences between the astaxanthin-producing transgenic plants generated using the different constructs as well as between the astaxanthin-accumulating and non-accumulating lines. Four developmental parameters were measured in the T1 *N. benthamiana* plants grown under greenhouse conditions, these parameters were: height, diameter of the canopy, flower number, and dry weights of the plants. At 4 weeks after planting, there was no significant difference in plant height between plants with HBFD1-CBFD2 and the non-astaxanthin accumulating plants, however the other two astaxanthin producing constructs produced significantly shorter plants. At 8 weeks, the non-transformed plants were the tallest and crtW and Cit/crtW plants were significantly the shortest (Table 3).

**Table 3:** Height of T1 *N. benthamiana* plants at 4, 6 and, 8 weeks after planting grown under greenhouse conditions.

	**Week 4**		**Week 6**		**Week 8**	
**Construct**	**Height (cm)**	**SG^a^**	**Height (cm)**	**SG**	**Height (cm)**	**SG**
pCAMBIA2201-crtW	7.0 ± 1.8	b	12.5 ± 3.5	b	19.6 ± 4.0	c
pCAMBIA2201-Cit/crtW	7.2 ± 2.3	b	12.5 ± 4.3	b	19.5 ± 4.6	c
pCAMBIA2201-HBFD1-CBFD2	8.2 ± 1.9	a	13.9 ± 3.6	b	22.1 ± 4.5	b
pCAMBIA2201	8.9 ± 2.8	a	15.9 ± 5.0	a	23.8 ± 4.2	ab
Non-transformed	8.3 ± 2.2	a	15.8 ± 5.7	a	24.1 ± 5.2	a

a *SG, significance group*.

*Values are means ± standard error of 90 replicates. Significance groups between experimental lines were determined by Tukey's HSD using variance calculated with ANOVA. α = 0.05*.

At 4, 6, and 8 weeks after planting the canopy diameter of the plants transformed with HBFD1-CBFD2 were the largest, however they shared significance with the crtW plants at 6 and 8 weeks (Supplementary Table S2). Regarding flower number, at 4 and 8 weeks there was no significant difference between any of the constructs or the non-transformed plants (Supplementary Table S3). The dry weight (Table 4), which was only measured at 8 weeks, ranged from 0.81 g in plants expressing Cit/crtW to 0.96 g in non-transformed plants. HBFD1-CBFD2 and crtW lines were not significantly different from the non-transformed lines. However, plants expressing Cit/crtW had the lowest dry weight, which was also significantly lower than the non-transformed plants (Table 4).

**Table 4:** Dry weight and water content of T1 *N. benthamiana* plants at 8 weeks after planting grown under greenhouse conditions.

**Construct**	**Dry weight (g)**	**SG^**a**^**	**Water content**
pCAMBIA2201-crtW	0.84 ± 0.3	ab	85.7%
pCAMBIA2201-Cit/crtW	0.81 ± 0.4	b	86.6%
pCAMBIA2201-HBFD1-CBFD2	0.94 ± 0.3	ab	85.1%
pCAMBIA2201	0.93 ± 0.4	ab	83.9%
Non-transformed	0.96 ± 0.4	a	84.0%

a *SG, significance group*.

*Values are means ± standard error of 90 replicates. Significance groups between experimental lines were determined by Tukey's HSD using variance calculated with ANOVA. α = 0.05*.

### UV Mortality Assay

*N. benthamiana* seedlings were placed under a UV lamp (fluence rate of 422 μmol/m^2^s) to determine whether plants accumulating astaxanthin had a differential tolerance to excessive UV exposure. Ten minutes of exposure was sufficient to produce significant differences in mortality rate between the astaxanthin-accumulating and non-accumulating plants with the non-transformed line having 27.0% mortality and all astaxanthin-accumulating lines having a mortality rate below 17% (Table 5). At every timepoint, the astaxanthin-accumulating plants had lower mortality rates than the non-transformed control. The LD_50_ for the non-transformed plants was 12.5 min, which was lower than most astaxanthin-accumulating lines. At 15 min, the two lines with highest astaxanthin accumulation, HBFD1-CBFD2 1-8 and 2-5, exhibited the lowest mortality rate at 57.7 and 60.3%, respectively, with line 1-8 having significantly lower rates than all other lines. There was a strong negative Pearson's correlation coefficient between astaxanthin content and morality at 10 min (ρ = −0.77), at 12.5 min (ρ = −0.82), and at 15 min (ρ = −0.75).

**Table 5:** Mortality rate of T1 *N. benthamiana* plants exposed to excessive UV radiation (fluence rate of 422 μmol/m^2^s).

		**10 min**		**12.5 min**		**15 min**	
**Construct**	**Line**	**Mortality rate**	**Significance group**	**Mortality rate**	**Significance group**	**Mortality rate**	**Significance group**
pCAMBIA2201-crtW	1-1	16.4%	b	44.4%	bc	67.6%	ab
pCAMBIA2201-crtW	1-10	14.9%	b	40.9%	bcd	65.8%	ab
pCAMBIA2201-Cit/crtW	2-4	9.0%	b	43.5%	bc	68.3%	ab
pCAMBIA2201-Cit/crtW	2-5	13.8%	b	42.2%	bc	71.6%	ab
pCAMBIA2201-Cit/crtW	2-16	13.8%	b	48.1%	ab	66.2%	ab
pCAMBIA2201-HBFD1-CBFD2	1-8	9.2%	b	37.2%	cd	57.7%	b
pCAMBIA2201-HBFD1-CBFD2	2-5	7.4%	b	35.1%	d	60.3%	ab
pCAMBIA2201-HBFD1-CBFD2	2-6	16.6%	ab	50.5%	ab	68.9%	ab
Non-transformed	–	27.0%	a	56.0%	a	79.4%	a

### Oxidative Burst Assay

**Figure 4:**
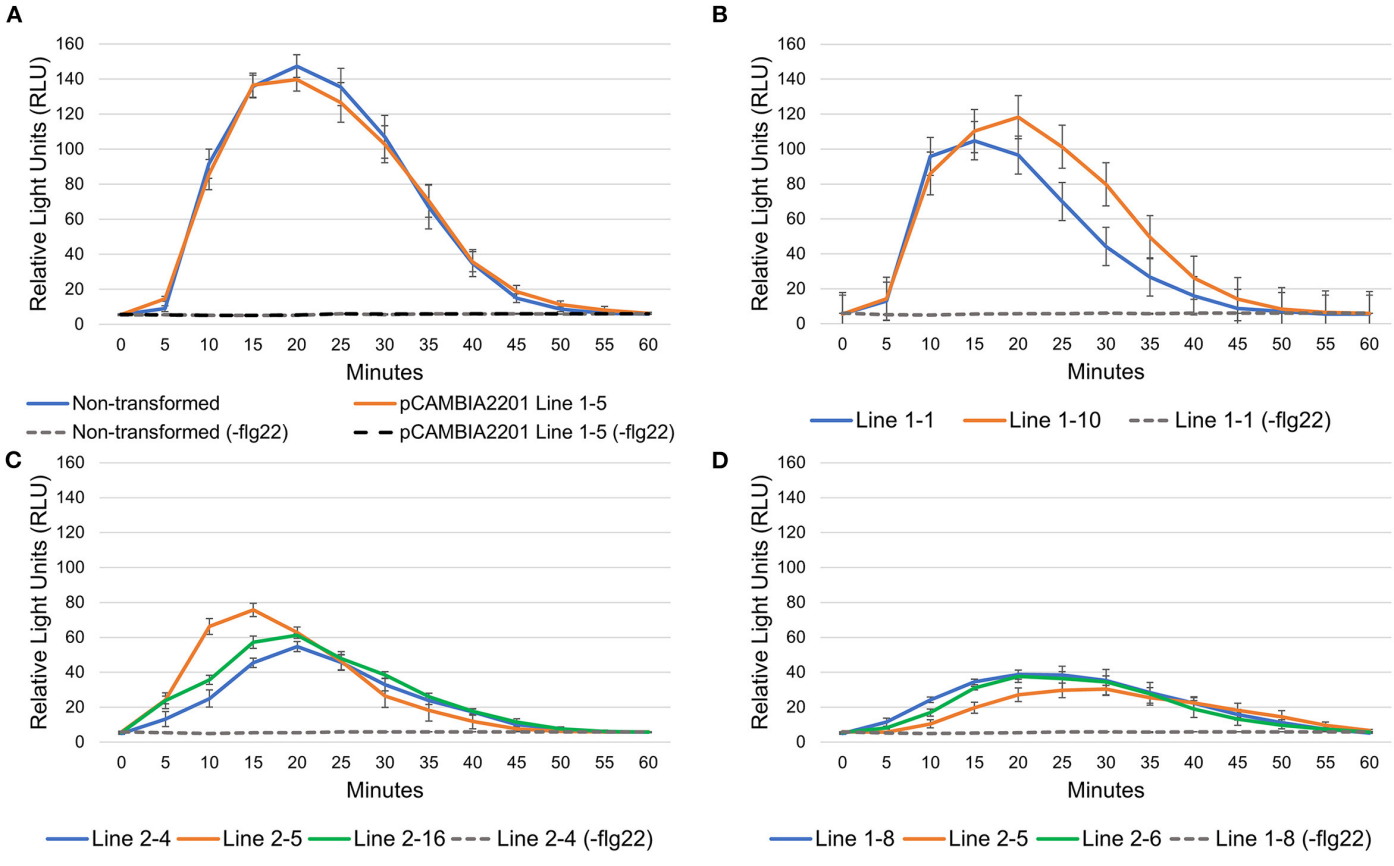
Flg22-triggered oxidative burst assays in T1 *N. benthamiana* leaf disks. The luminescence readings are from **(A)** empty vector and non-transformed control plants, **(B)** plants expressing crtW, **(C)** plants expressing Cit/crtW, and **(D)** plants expressing HBFD1-CBFD2. Assay solution containing 500 nM flg22 was added to the leaf disks at 0 min and luminescence measurements started immediately. For each sample analyzed, a control sample was included containing no flg22 (-flg22) in the assay mix, which is represented by the dashed lines on each chart.

Astaxanthin has a higher antioxidant potential than most other carotenoids, thus an assay was performed to determine whether astaxanthin-accumulating plants had a differential response to the induction of ROS. Plants produce ROS upon recognition of pathogen-associated molecular patterns (PAMPs), which are molecular motifs conserved across similar pathogens and can consist of a peptide, nucleic acid, cell membrane component or other pathogen-related molecule (Silhavy et al., 2010; Dammermann et al., 2013; Mahla et al., 2013). As a form of plant defense, when plant cell receptors detect a PAMP, a defense response is elicited to protect the plant from the invading pathogen (Mogensen, 2009). A well-studied PAMP is bacterial flagellin. Specifically, a 22-amino acid conserved motif of the flagellin peptide, which is the primary component of the bacterial flagellum, is detected by the plant and initiates PAMP-triggered immunity response (Felix et al., 1999). An assay was performed using the conserved motif of the flagellin peptide, flg22, to elicit the oxidative burst defense response in astaxanthin-accumulating plants (Figure 4). All astaxanthin-accumulating transgenic lines had lower ROS levels than the non-astaxanthin accumulating lines. The average peak of the relative light unit (RLU) readings for both non-astaxanthin accumulating plants, the non-transformed and pCAMBIA2201 empty vector, was around 140 RLUs at 20 min. Plants expressing HBFD1-CBFD2 had the lowest RLUs readings compared to all other constructs with peaks ranging from 30 to 40 RLUs, most likely due to having the highest astaxanthin levels (Figure 4).

### Plant Transformation Without Antibiotic Selection

Due to the distinct color difference observed in the astaxanthin and non-astaxanthin accumulating *N. benthamiana* plants, an experiment was performed to determine whether it was possible to recover transformed plants without the use of antibiotic selection. The reasoning was that constructs without the β-glucuronidase (*GUS*) and neomycin phosphotransferase II (*NPTII*) genes could be created for the generation of astaxanthin-producing transgenic plants, eliminating these potentially undesirable genes from the regenerated plants. As expected, the transformation efficiency was lower without antibiotic selection compared to using kanamycin in the media, ranging from 4 to 8% with no selection compared to 21–25% with selection (Table 1), however, it was still possible to easily identify the transgenic plants due to their distinct coloration. Construct pCAMBIA2201-HBFD1-CBFD2 had the highest transformation efficiency both with and without selection at 25 and 8%, respectively. Also as expected, the total number of regenerated shoots was higher in media without selection due to a larger number of escapes. The total number of shoots produced on selection media with pCAMBIA2201-crtW (76 total shoots) and pCAMBIA2201 (77 total shoots) were lower than the other constructs due to certain leaf disks not producing any shoots (Table 1), which was also observed in the previously described *N. benthamiana* transformations.

## Discussion

In this study we demonstrated that the genes coding for two enzymes, HBFD1 and CBFD2, from the genus *Adonis* serve as an alternative method for producing astaxanthin in transgenic *N. benthamiana*. These two enzymes have been well-studied and their ability to convert β-carotene into astaxanthin was previously confirmed through bacterial complementation and biochemical assays, however their function as an alternative route to astaxanthin biosynthesis in heterologous plant species had not been previously evaluated (Cunningham and Gantt, 2011). We found that plants transformed with these two genes accumulated astaxanthin at levels similar to previous studies (Table 2; Supplementary Table S4) (Zhong et al., 2011; Huang et al., 2013), the plant's phenotype was minimally affected, and in most cases the plants with the *A. aestivalis* biosynthetic pathway grew more similar to the non-transformed plants than the plants with the *Brevundimonas* sp. SD212 β-carotene ketolase enzyme (Tables 3, 4; Supplementary Tables S2, S3).

The color of the transgenic *N. benthamiana* was similar between transgenic lines and constructs. However, the quantification of astaxanthin showed that the two lines that accumulated the highest amounts were those transformed with HBFD1/CBFD2. Line HBFD1/CBFD2 2-5 accumulated 2.5–7.5 times more astaxanthin compared on a dry weight basis to the lines transformed with the other two constructs. These results suggest the HBFD1/CBFD2 construct was more effective in converting astaxanthin from β-carotene. Alternatively, differences in promoter structure and expression could also result in the observed differences. It is also of note that regardless of the amount of astaxanthin, there were no significant differences in total carotenoid accumulation (Supplementary Table S1) suggesting that the conversion of the endogenous carotenoids into astaxanthin has little or no effect on the regulation or production of total carotenoids. Previous research has reported conflicting results in relation to astaxanthin production and total carotenoid accumulation. Studies in *Nicotiana glauca* transformed with a similar *crtW* as well as *Solanum lycopersicum* transformed with *crtW* and bacterial β-carotene hydroxylase (*crtZ*) from *Brevundimonas* sp. SD212 showed that the astaxanthin-producing transgenic lines accumulated less overall carotenoids in mature leaves (Mortimer et al., 2017; Nogueira et al., 2017). However, two other studies, one with a plastid transformed *Nicotiana tabacum* with *crtW* and *crtZ* and another with *Solanum lycopersicum* transformed with algal β-carotene ketolase (*BKT*) from *Chlamydomonas reinhardtii* and algal β-carotene hydroxylase (*BHY*) from *Haematoccocus pluvialis* showed that those astaxanthin-accumulating plants had a 2- to 5-fold increase in total carotenoids in the astaxanthin-accumulating lines (Hasunuma et al., 2008; Huang et al., 2013). This could be due to differences in the transformation types, genes used, or species that were transformed, but in all cases, there was variability in the ratio of total carotenoids between controls and astaxanthin-accumulating plants.

Previous observations have determined that the amount of astaxanthin that can be produced in transgenic tissue is not consistent. Reported quantities of astaxanthin range from 0.2 μg/g of dry weight in *Solanum tuberosum* tubers through nuclear transformation, up to 5.44 mg/g of dry weight in *Nicotiana tabacum* leaves through plastid transformation (Morris et al., 2006; Hasunuma et al., 2008) (Supplementary Table S4). The exact genes and promoters used, the plant system being transformed, tissue analyzed, and transformation type (nuclear/plastid) all could potentially impact the amount of astaxanthin produced. In this study, a line of *N. benthamiana* plants possessing HBFD1 and CBFD2 produced 0.99 mg/g of astaxanthin in dry weight leaf tissue (Table 2). Therefore, the constructs used here will be useful to engineer astaxanthin accumulation in various tissues including fruit of the plants as the chimeric genes are driven by constitutive promoters. In support of this, the flowers from every construct-expressing plant had a light pinkish-copper coloration compared to white petals of the non-astaxanthin-accumulating *N. benthamiana* plants and the tissue of the transgenic seed pods had the same copper-colored phenotype as the vegetative tissue. The actual seeds, however, were too dark in appearance to have a visually different phenotype. There are three other reports of transgenic astaxanthin producing plants that produced greater amounts than this line; 5.44 mg/g of dry weight in *Nicotiana tabacum* leaves through plastid transformation with the *crtW* gene from *Brevundimonas* sp. SD212, 3.12 mg/g of dry weight in *Solanum lycopersicum* fruit through nuclear transformation with BKT from *Chlamydomonas reinhardtii*, and 2.07 mg/g of dry weight in *Arabidopsis thaliana* leaves through nuclear transformation with the *BKT* gene from *Chlamydomonas reinhardtii* (Hasunuma et al., 2008; Zhong et al., 2011; Huang et al., 2013) (Supplementary Table S4). There were eighteen different studies listed in Supplementary Table S4 that compared astaxanthin content in transgenic plants. There was over a 20,000-fold difference in the astaxanthin content between those studies, with a median value of only 91.60 μg/g of dry weight and in the experimental lines from this study the lowest producing line had 130 μg/g of dry weight (Table 2). As shown from these experiments, plants expressing both *HBFD1* and *CBFD2* can produce levels of astaxanthin higher than most of the studies that use the *crtW* gene. As evidenced from the data produced in this study, plants with both *HBFD1* and *CBFD2* had higher levels of astaxanthin than either the native or codon-optimized versions of *crtW* and they also had lower levels of canthaxanthin, suggesting that these enzymes are more efficient at converting β-carotene into astaxanthin. Additionally, between the crtW and Cit/crtW constructs, plants with Cit/crtW accumulated less canthaxanthin in all tested lines compared to crtW plants. Two out of three Cit/crtW lines also had 2.5–3.5 times more β-carotene than the crtW lines. This suggests that the Cit/crtW construct was more efficient at converting β-carotene into astaxanthin.

When looking at all the carotenoids that were quantified, astaxanthin and β-carotene combined made up around 30–50% of the total carotenoids in most lines, however in the crtW lines and one of the Cit/crtW lines, astaxanthin and β-carotene only made up 15–20%, which could be due to inefficient conversion of β-carotene into astaxanthin, causing an accumulation of ketolated intermediates. We did not analyze other ketolated intermediates besides canthaxanthin in this experiment, therefore this hypothesis is only speculative. In previous astaxanthin transformation studies, when ketocarotenoid intermediates were measured, canthaxanthin which is a 4,4'-keto-β,β-carotene is usually the most prevalent intermediate (Hasunuma et al., 2008; Mortimer et al., 2017; Nogueira et al., 2017), however in some cases echinenone was most prevalent, which is a 3-hydroxy-4'-keto-β,β-carotene (Mortimer et al., 2016). There is also the potential for other endogenous carotenoids to be ketolated by a β-carotene ketolase enzyme, such as a 4-ketozeaxanthin, 4-ketolutein, or 4-ketoantheraxanthin (Gerjets et al., 2007; Mortimer et al., 2017; Nogueira et al., 2017).

The carotenoid composition may pose some impact on photoprotection or some other unknown effect as it relates to growth. Some of the astaxanthin-accumulating transgenic plants showed impacted growth relative to the non-transformed plants. Height and dry weight were significantly less in the plants harboring crtW and Cit/crtW, and both of their flowering was significantly delayed (Tables 3, 4; Supplementary Tables S3). The plants expressing the *Adonis* genes, however, were no different from non-transformed plants in flowering or dry weight and were not significantly different in height than the empty vector control. The plants with the *Adonis* genes had the highest levels of astaxanthin and highest levels of β-carotene accumulation on average compared to plants containing the other constructs. This suggests that these enzymes were converting β-carotene into astaxanthin more efficiently and affecting the carotenoid composition the least. Further research could be performed using these plants to determine whether photosynthesis or the photoprotection from carotenoids is being directly affected by the biosynthesis of astaxanthin which is altering the natural composition of carotenoids.

Astaxanthin has a higher antioxidant potential compared to the carotenoids in the core carotenoid pathway due to the additional oxygen containing functional groups (Higuera-Ciapara et al., 2006). Plants accumulating astaxanthin have the potential to have increased antioxidation properties. To test this hypothesis, plants were given an intense dose of UV exposure to determine whether the increased ROS would differentially affect plants with and without astaxanthin. The results showed that the mortality rates in all astaxanthin-accumulating lines were lower than all the non-astaxanthin accumulating lines (Table 5). Specifically, the two highest accumulating astaxanthin lines, expressing HBFD1-CBFD2, had the two lowest mortality rates in each treatment. Overall, mortality rates showed a significant negative correlation (at 10 min ρ = −0.77, at 12.5 min ρ = −0.82, and at 15 min ρ = −0.75) with astaxanthin content for all the lines, providing evidence for the ability of astaxanthin to protect against intense UV exposure. These results are consistent with a previous study in astaxanthin-accumulating transgenic tomatoes that had a 3- to 5-fold increase in antioxidizing capabilities compared to the controls (Huang et al., 2013).

UV exposure is not the only mechanism that can cause a buildup of ROS in plants though. As a defense response, plants will elicit an oxidative burst when detecting certain pathogen-associated molecular patterns to kill the invading pathogen before it can cause any harm to the plant. This is an important plant defense mechanism for tolerance to certain pathogens. A ROS accumulation assay was performed using the flagellin peptide, flg22, which elicits an oxidative burst in *N. benthamiana*. Like the UV exposure assay, it was found that the amount of ROS was lessened in the astaxanthin-accumulating plants when elicited by the peptide. This could have drawbacks in terms of pathogen susceptibility, however there was no obvious difference when growing all the plants in the greenhouse. Further research is needed to determine whether pathogen susceptibility was affected in plants that accumulate astaxanthin. In addition, differential astaxanthin accumulation may serve as a template for studying pathogen susceptibility thresholds due to the proportionate amount of astaxanthin accumulation to ROS mitigation measured in these plants.

In addition to the physiological effects that astaxanthin accumulation had on the plants, it had a prominent effect on the coloration of the plant. Since this copper-color was so easily visualized and consistently present throughout all growth stages, it could be useful as a scorable marker in transformation experiments. Most transformation experiments require a plant to be transformed with a constitutively expressed marker gene, such as *GUS* or a fluorescent protein, that serve no purpose other than to recover transgenic tissue. To identify transgenic tissue, most of these marker genes also require either specialized equipment or involve an assay that destroys tissue. However, astaxanthin accumulation does not require any sort of equipment or assay to test for its presence and it is easily observed with the naked eye in regenerating tissue. Additionally, the coloration allows for the easy differentiation of chimeric tissue. This led to an experiment transforming *N. benthamiana* without the use of antibiotic selection. The use of antibiotic selection allows for a higher transformation efficiency, greatly reducing the amount of screening needed to identify and recover transgenic tissue. This study found that identifying and recovering transgenic plants based solely on color from astaxanthin accumulation was possible and did not add any extra effort in the screening process compared to using antibiotic selection. This means that astaxanthin production, using the *Adonis* or the bacterial genes, could substitute other scorable markers in transformation experiments by providing a more efficient transgenic tissue screening process, negligible effects on growth, and a trait that provides a higher level of protection from UV in the plants. Additionally, the *Adonis*-derived genes, which are of plant origin, could form part of a construct with solely plant-based genes.

Two lines harboring HBFD1-CBFD2 accumulated higher levels of astaxanthin than plants transformed with either of the two other constructs. The *A. aestivalis* gene-derived construct had the least impact on the growth and development of *N. benthamiana* plants. The plants with crtW and Cit/crtW had very similar results in most of the assays performed, however most of the lines with Cit/crtW had less canthaxanthin and more β-carotene supporting that the plant codon-optimization was beneficial. In conclusion, the enzymes responsible for the astaxanthin production in *A. aestivalis* serve as an alternative route to astaxanthin biosynthesis when transformed into *N. benthamiana*. They also were superior in their ability to produce astaxanthin and pose minimal affects when compared to the bacterial β-carotene ketolase, *crtW*.

## Materials and Methods

### Cloning and Plant Transformation

The first construct combined *HBFD1* and *CBFD2* from *Adonis* (obtained from the Addgene plasmids 53564 and 53574, Addgene, MA, USA). The two genes were subcloned into a plasmid in front of a bidirectional constitutive promoter consisting of a full-length CaMV 35S and a minimal FMV 34S promoter along with the Nopaline synthase (NOS) and CaMV 35S terminators, respectively. The genes, along with the promoter and terminators, were subsequently subcloned into pCAMBIA2201. The resulting plasmid was designated as pCAMBIA2201-HBFD1-CBFD2 (Figure 2A). The second and third constructs contained either a native or a plant codon-optimized (Ahmad et al., 2013; Xu et al., 2013) version of the *crtW* from *Brevundimonas* sp. SD212 (GenBank AB181388) with the predicted *N*-terminal transit peptide from the small subunit of Rubisco (SSU) from *Citrus sinensis* (GenBank XM_006482191). Both chimeric genes were synthesized (GenScript Biotech, NJ, USA) and subcloned between the FMV 34S promoter and NOS terminator. Both genes were subsequently subcloned into pCAMBIA2201 resulting in plasmid pCAMBIA2201-crtW (native) and pCAMBIA2201-Cit/crtW (codon-optimized) (Figures 2B,C). At each step of cloning, the sequences were confirmed through Sanger sequencing. After verifying the sequences, the constructs were transformed into *Agrobacterium tumafaciens* strain Agl1.

Six-week-old, aseptically grown *N. benthamiana* plants were used for leaf disk transformations (Horsch et al., 1989) with modifications. Briefly, leaf disks were punched from fully expanded leaves and placed adaxial side down on Murashige and Skoog (MS) media (full strength MS basal medium salts with 3% sucrose, 2 mg/L 6-benzylaminopurine (BA), and 0.6% (w/v) agar, pH 5.8) at a density of 10 disks per 10 cm plate. Subsequently, 75 μL of *Agrobacterium* suspension was pipetted onto the abaxial side of each leaf, the leaf disks were placed in the dark for 48 h at 22°C and then transferred to MS media supplemented with 100 μg/mL of kanamycin and 500 μg/mL of cefotaxime and were placed in a culture room with 16-h photoperiod under grow lights with 150 μmol/m^2^s photon flux density.

### Primer and PCR Conditions

PCR reactions with primer pairs specific to each carotenogenic transgene (Supplementary Table S5) were used to verify the presence of the transgenes. Genomic DNA was extracted from fresh or frozen *N. benthamiana* leaves using the DNeasy Plant Mini Kit (Qiagen, Hilden, Germany). Amplification was performed at 95°C for 2 min, followed by 30 cycles at 95°C for 30 s, 55.3°C for 30 s, and 72°C for 1 min per kilobase, followed by 72°C for 5 min. PCR products were visualized on an 0.8% agarose gel stained with GelRed Nucleic Acid Gel Stain (Biotium, CA, USA) using a UV transilluminator.

### Plant Growth Conditions

Plants were grown under greenhouse conditions, with photosynthetically active radiation measuring between 1,100 and 1,400 μmol/m^2^s while the plants were being evaluated. To evaluate the growth rate, T1 *N. benthamiana* seeds were planted and grown under greenhouse conditions. Flower number, height, and canopy diameter of the plants were recorded at 4, 6, and 8 weeks after planting. Canopy diameter of the plant was recorded by measuring the longest distance from leaf tip to leaf tip and then measuring the perpendicular distance and averaging the two numbers. At 8 weeks, data on the fresh weight and dry weight for each plant was recorded. The trial was performed in a randomized complete block design with three transgenic lines from each construct and ten technical replicates from each transgenic line and the entire experiment was repeated a total of three times. The data presented consists of the average of the three experiments. The negative controls for this experiment included non-transformed plants and T1 pCAMBIA2201 empty vector plants. Each repeated experiment contained 150 plants (ten technical replicates from 15 lines) arranged in a 10 × 15 grid with each pot spaced 8 cm from the next closest pot.

### Carotenoid Extraction and Quantification Through Liquid Chromatography/Mass Spectrometry

For the carotenoid extractions, frozen leaf tissue was weighed and manually ground in a solvent solution of water, methanol, and chloroform (1:1:4 ratio). After extraction, the organic layer was drawn off in glass vials, protected from light and stored at −20°C until used for LC/MS. The extracted samples were first separated via a reverse phase gradient Acclaim C30 column in a Thermo Scientific Ultimate 3000 HPLC system. The mobile phases used for separation were water with 0.1% formic acid (mobile phase A) and methanol with 0.1% formic acid (mobile phase B). After separation in the C30 column, a mass spectrometer further analyzed the samples by characterizing mass peaks and collision induced dissociation breakdown products using a ThermoScientific LTQ XL linear quadrupole ion trap mass spectrometer with ESI in positive ion mode operating with XCALIBUR 2.2 SP1.48. Carotenoids were identified based on their retention time and mass:charge (m/z) peaks. Standard curves from each authentic analytic standard were used for carotenoid quantitation.

### UV Mortality Assay

T1 *N. benthamiana* seeds were sterilized and cultured on agar media and after 10 days, the seedlings were exposed to a UV lamp with a fluence rate of 422 μmol/m^2^s. The plants were divided into three treatments of 10, 12.5, and 15 min of UV exposure and then were returned to growth chamber and received 285 μmol/m^2^s of photosynthetically active radiation with a 16-h photoperiod. The plates were scored for mortality at 96 h.

### Oxidative Burst Assay

Measurement of reactive oxygen-species generation was performed as described by Boutrot et al. (2010), using the conserved motif of the flagellin peptide, flg22, to elicit an oxidative burst defense response in 1.5 mm *N. benthamiana* leaf disks. Luminol and horseradish peroxidase were used to quantify the magnitude of the oxidative burst. The luminescence was measured using a BioTek Synergy 2 plate reader using the Gen5.1.11 software.

## Data Availability Statement

The original contributions presented in the study are included in the article/Supplementary Material, further inquiries can be directed to the corresponding author.

## Author Contributions

QA carried out the experiments, data collection, and took the lead in writing the manuscript. QA and VF prepared the manuscript for submission. JC and BR revised the final manuscript. JC conceived the original idea. All authors were involved in conceptualizing and designing the experiments, and the analysis and interpretation of results and contributed to the article and approved the submitted version.

## Funding

This work was funded by the UF/IFAS Plant Breeding Graduate Initiative and was supported by the University of Florida Mass Spectrometry Research and Education Center (NIH S10 OD021758-01A1).

## Conflict of Interest

The authors declare that the research was conducted in the absence of any commercial or financial relationships that could be construed as a potential conflict of interest.

## Publisher's Note

All claims expressed in this article are solely those of the authors and do not necessarily represent those of their affiliated organizations, or those of the publisher, the editors and the reviewers. Any product that may be evaluated in this article, or claim that may be made by its manufacturer, is not guaranteed or endorsed by the publisher.

## References

[B1] Ahmad T. Sablok G. Tatarinova T. V. Xu Q. Deng X.-X. Guo W.-W. (2013). Evaluation of codon biology in citrus and *Poncirus trifoliata* based on genomic features and frame corrected expressed sequence tags. DNA Res. 20, 135–150. 10.1093/dnares/dss039 23315666 PMC3628444

[B2] Boutrot F. Segonzac C. Chang K. N. Qiao H. Ecker J. R. Zipfel C.. (2010). Direct transcriptional control of the Arabidopsis immune receptor FLS2 by the ethylene-dependent transcription factors EIN3 and EIL1. Proc. Natl. Acad. Sci. U. S. A. 107, 14502–14507. 10.1073/pnas.1003347107 20663954 PMC2922558

[B3] Cunningham F. X. Gantt E. (2005). A study in scarlet: enzymes of ketocarotenoid biosynthesis in the flowers of Adonis aestivalis. Plant J. 41, 478–492. 10.1111/j.1365-313X.2004.02309.x 15659105

[B4] Cunningham F. X. Gantt E. (2011). Elucidation of the pathway to astaxanthin in the flowers of Adonis aestivalis. Plant Cell 23, 3055–3069. 10.1105/tpc.111.086827 21862704 PMC3180810

[B5] Dammermann W. Wollenberg L. Bentzien F. Lohse A. Lüth S. (2013). Toll like receptor 2 agonists lipoteichoic acid and peptidoglycan are able to enhance antigen specific IFNγ release in whole blood during recall antigen responses. J. Immunol. Methods 396, 107–115. 10.1016/j.jim.2013.08.004 23954282

[B6] Felix G. Duran J. D. Volko S. Boller T. (1999). Plants have a sensitive perception system for the most conserved domain of bacterial flagellin. Plant J. 18, 265–276. 10.1046/j.1365-313X.1999.00265.x 10377992

[B7] Fraser P. D. Miura Y. Misawa N. (1997). *In vitro* characterization of astaxanthin biosynthetic enzymes. J. Biol. Chem. 272, 6128–6135. 10.1074/jbc.272.10.6128 9045623

[B8] Gerjets T. Sandmann M. Zhu C. Sandmann G. (2007). Metabolic engineering of ketocarotenoid biosynthesis in leaves and flowers of tobacco species. Biotechnol. J. 2, 1263–1269. 10.1002/biot.200700040 17619231

[B9] Harada H. Maoka T. Osawa A. Hattan J. I. Kanamoto H. Shindo K.. (2014). Construction of transplastomic lettuce (*Lactuca sativa*) dominantly producing astaxanthin fatty acid esters and detailed chemical analysis of generated carotenoids. Transgenic Res. 23, 303–315. 10.1007/s11248-013-9750-3 24287848

[B10] Hasunuma T. Miyazawa S. I. Yoshimura S. Shinzaki Y. Tomizawa K. I. Shindo K.. (2008). Biosynthesis of astaxanthin in tobacco leaves by transplastomic engineering. Plant J. 55, 857–868. 10.1111/j.1365-313X.2008.03559.x 18494855

[B11] Higuera-Ciapara I. Félix-Valenzuela L. Goycoolea F. M. (2006). Astaxanthin: a review of its chemistry and applications. Crit. Rev. Food Sci. Nutr. 46, 185–196. 10.1080/10408690590957188 16431409

[B12] Horsch R. B. Fry J. Hoffmann N. Neidermeyer J. Rogers S. G. Fraley R. T. (1989). “Leaf disc transformation,” in Plant Molecular Biology Manual (Dordrecht: Springer), 63–71.

[B13] Huang J. C. Zhong Y. J. Liu J. Sandmann G. Chen F. (2013). Metabolic engineering of tomato for high-yield production of astaxanthin. Metab. Eng. 17, 59–67. 10.1016/j.ymben.2013.02.005 23511430

[B14] Johnson E. A. An G. H. (1991). Astaxanthin from microbial sources. Crit. Rev. Biotechnol. 11, 297–326. 10.3109/07388559109040622

[B15] Liu H. Zhang C. Zhang X. Tan K. Zhang H. Cheng D.. (2020). A novel carotenoids-producing marine bacterium from noble scallop *Chlamys nobilis* and antioxidant activities of its carotenoid compositions. Food Chem. 320:126629. 10.1016/j.foodchem.2020.126629 32203829

[B16] Mahla R. S. Reddy M. C. Vijaya Raghava Prasad D. Kumar H. (2013). Sweeten PAMPs: role of sugar complexed PAMPs in innate immunity and vaccine biology. Front. Immunol. 4:248. 10.3389/fimmu.2013.00248 24032031 PMC3759294

[B17] Mogensen T. H.. (2009). Pathogen recognition and inflammatory signaling in innate immune defenses. Clin. Microbiol. Rev. 22, 240–273. 10.1128/CMR.00046-08 19366914 PMC2668232

[B18] Morris W. L. Ducreux L. J. M. Fraser P. D. Millam S. Taylor M. A. (2006). Engineering ketocarotenoid biosynthesis in potato tubers. Metab. Eng. 8, 253–263. 10.1016/j.ymben.2006.01.001 16542864

[B19] Mortimer C. L. Misawa N. Ducreux L. Campbell R. Bramley P. M. Taylor M.. (2016). Product stability and sequestration mechanisms in *Solanum tuberosum* engineered to biosynthesize high value ketocarotenoids. Plant Biotechnol. J. 14, 140–152. 10.1111/pbi.12365 25845905 PMC11388937

[B20] Mortimer C. L. Misawa N. Perez-Fons L. Robertson F. P. Harada H. Bramley P. M.. (2017). The formation and sequestration of nonendogenous ketocarotenoids in transgenic Nicotiana glauca. Plant Physiol. 173, 1617–1635. 10.1104/pp.16.01297 28153925 PMC5338661

[B21] Nogueira M. Enfissi E. M. A. Martínez Valenzuela M. E. Menard G. N. Driller R. L. Eastmond P. J.. (2017). Engineering of tomato for the sustainable production of ketocarotenoids and its evaluation in aquaculture feed. Proc. Natl. Acad. Sci. U. S. A. 114, 10876–10881. 10.1073/pnas.1708349114 28973873 PMC5642710

[B22] O'Connor I. O'Brien N. (1998). Modulation of UVA light-induced oxidative stress by β-carotene, lutein and astaxanthin in cultured fibroblasts. J. Dermatol. Sci. 16, 226–230. 10.1016/S0923-1811(97)00058-3 9651820

[B23] Pierce E. C. LaFayette P. R. Ortega M. A. Joyce B. L. Kopsell D. A. Parrott W. A. (2015). Ketocarotenoid production in soybean seeds through metabolic engineering. PLoS ONE 10:e0138196. 10.1371/journal.pone.0138196 26376481 PMC4574205

[B24] Silhavy T. J. Kahne D. Walker S. (2010). The bacterial cell envelope. Cold Spring Harb. Perspect. Biol. 2:a000414. 10.1101/cshperspect.a000414 20452953 PMC2857177

[B25] Visser H. Van Ooyen A. J. J. Verdoes J. C. (2003). Metabolic engineering of the astaxanthin-biosynthetic pathway of Xanthophyllomyces dendrorhous. FEMS Yeast Res. 4, 221–231. 10.1016/S1567-1356(03)00158-2 14654426

[B26] Wei X. Chen C. Yu Q. Gady A. Yu Y. Liang G.. (2014). Novel expression patterns of carotenoid pathway-related genes in citrus leaves and maturing fruits. Tree Genet. Genomes 10, 439–448. 10.1007/s11295-013-0688-7

[B27] Xu C. Dong J. Tong C. Gong X. Wen Q. Zhuge Q. (2013). Analysis of synonymous codon usage patterns in seven different citrus species. Evol. Bioinform. Online 9, 215–228. 10.4137/EBO.S11930 23761955 PMC3667683

[B28] Zhong Y. J. Huang J. C. Liu J. Li Y. Jiang Y. Xu Z. F.. (2011). Functional characterization of various algal carotenoid ketolases reveals that ketolating zeaxanthin efficiently is essential for high production of astaxanthin in transgenic Arabidopsis. J. Exp. Bot. 62, 3659–3669. 10.1093/jxb/err070 21398427 PMC3130182

[B29] Zhu Q. Zeng D. Yu S. Cui C. Li J. Li H.. (2018). From golden rice to aSTARice: bioengineering astaxanthin biosynthesis in rice endosperm. Mol. Plant 11, 1440–1448. 10.1016/j.molp.2018.09.007 30296601

