# Supplementary material for: Engineering a Plant-Derived Astaxanthin Synthetic Pathway Into Nicotiana benthamiana
Source: Front Plant Sci. 2022 Jan 18;12:831785. doi: 10.3389/fpls.2021.831785 (PMC8804313; doi:10.3389/fpls.2021.831785)
Supplement: Supplementary file 1 [file Table_1.DOCX]

**Supplementary Table 1.** Quantification of zeaxanthin, lutein and total carotenoids in T1 *N. benthamiana* leaves using high performance liquid chromatography coupled with mass spectrometry. Values are means ± standard error of four replicates. Significance groups between experimental lines were determined by Tukey’s HSD using variance calculated with ANOVA. α = 0.05. Minimum detection limits for the compounds were 1 µg/g of dry tissue.

|  |  | Zeaxanthin | | | Lutein | | | Total carotenoids | |
| --- | --- | --- | --- | --- | --- | --- | --- | --- | --- |
| Construct | Line | mg/g of dry tissue | % of total carotenoids | SG^1^ | mg/g of dry tissue | % of total carotenoids | SG | mg/g of dry tissue | SG |
| pCAMBIA2201-crtW | 1-1 | 0.002 ± 0.000 | 0.06% | b | 0.027 ± 0.001 | 0.79% | a | 2.99 ± 0.67 | a |
| pCAMBIA2201-crtW | 1-10 | 0.003 ± 0.001 | 0.07% | ab | 0.040 ± 0.003 | 1.02% | a | 3.41 ± 0.66 | a |
| pCAMBIA2201-Cit/crtW | 2-4 | 0.003 ± 0.000 | 0.07% | ab | 0.020 ± 0.001 | 0.51% | a | 3.38 ± 0.78 | a |
| pCAMBIA2201-Cit/crtW | 2-5 | 0.005 ± 0.002 | 0.15% | ab | 0.039 ± 0.002 | 1.09% | a | 3.58 ± 0.07 | a |
| pCAMBIA2201-Cit/crtW | 2-16 | 0.003 ± 0.000 | 0.08% | ab | 0.033 ± 0.012 | 0.82% | a | 3.95 ± 0.04 | a |
| pCAMBIA2201-HBFD1-CBFD2 | 1-8 | 0.004 ± 0.001 | 0.11% | ab | 0.035 ± 0.002 | 1.03% | a | 3.36 ± 0.09 | a |
| pCAMBIA2201-HBFD1-CBFD2 | 2-5 | 0.005 ± 0.000 | 0.12% | ab | 0.022 ± 0.002 | 0.53% | a | 3.73 ± 0.53 | a |
| pCAMBIA2201-HBFD1-CBFD2 | 2-6 | 0.004 ± 0.002 | 0.15% | ab | 0.024 ± 0.004 | 0.96% | a | 2.70 ± 0.24 | a |
| pCAMBIA2201 | 1-5 | 0.006 ± 0.001 | 0.14% | ab | 0.029 ± 0.009 | 0.71% | a | 3.48 ± 0.78 | a |
| Non-transformed | - | 0.010 ± 0.002 | 0.26% | a | 0.027 ± 0.001 | 0.73% | a | 3.27 ± 0.59 | a |

^1^SG = Significance Group

**Supplementary Table 2.** Canopy diameter of T1 *N. benthamiana* plants at four, six and, eight weeks after planting grown under greenhouse conditions. Values are means ± standard error of 90 replicates. Significance groups between experimental lines were determined by Tukey’s HSD using variance calculated with ANOVA. α = 0.05.

|  | Week 4 | |  | Week 6 | |  | Week 8 | |  |
| --- | --- | --- | --- | --- | --- | --- | --- | --- | --- |
| Construct | Canopy Diameter (cm) | SG^1^ |  | Canopy Diameter (cm) | SG |  | Canopy Diameter (cm) | SG |  |
| pCAMBIA2201-crtW | 9.5 ± 1.9 | b |  | 15.9 ± 1.8 | ab |  | 16.0 ± 1.5 | ab |  |
| pCAMBIA2201-Cit/crtW | 9.7 ± 2.9 | b |  | 15.3 ± 2.1 | bc |  | 15.7 ± 1.3 | bc |  |
| pCAMBIA2201-HBFD1-CBFD2 | 11.1 ± 3.0 | a |  | 16.3 ± 2.1 | a |  | 16.5 ± 1.6 | a |  |
| pCAMBIA2201 | 11.2 ± 3.2 | a |  | 15.0 ± 1.6 | c |  | 15.6 ± 1.6 | bc |  |
| Non-transformed | 10.5 ± 2.5 | ab |  | 15.0 ± 2.0 | c |  | 15.3 ± 1.3 | c |  |

^1^SG = Significance Group.

**Supplementary Table 3.** Number of flowers per T1 *N. benthamiana* plant at four, six and, eight weeks after planting grown under greenhouse conditions. Values are means ± standard error of 90 replicates. Significance groups between experimental lines were determined by Tukey’s HSD using variance calculated with ANOVA. α = 0.05.

|  | Week 4 | |  | Week 6 | |  | Week 8 | |  |
| --- | --- | --- | --- | --- | --- | --- | --- | --- | --- |
| Construct | Flowers per plant | SG^1^ |  | Flowers per plant | SG |  | Flowers per plant | SG |  |
| pCAMBIA2201-crtW | 0.4 ± 0.5 | a |  | 3.3 ± 2.5 | b |  | 11.2 ± 5.0 | a |  |
| pCAMBIA2201-Cit/crtW | 0.5 ± 0.5 | a |  | 3.4 ± 2.8 | b |  | 11.7 ± 5.7 | a |  |
| pCAMBIA2201-HBFD1-CBFD2 | 0.5 ± 0.5 | a |  | 3.9 ± 2.2 | ab |  | 12.5 ± 5.0 | a |  |
| pCAMBIA2201 | 0.5 ± 0.5 | a |  | 5.1 ± 3.3 | a |  | 12.7 ± 4.2 | a |  |
| Non-transformed | 0.5 ± 0.5 | a |  | 4.8 ± 3.4 | a |  | 12.6 ± 5.1 | a |  |

^1^SG = Significance Group.

**Supplementary Table 4.** Reported accumulation of astaxanthin in genetically modified plants.

| Plant Species | Transgenes used | Origin of transgenes used | Promoter used | Type of transformation | Astaxanthin per dry weight of tissue | % of total carotenoids | Tissue | Source |
| --- | --- | --- | --- | --- | --- | --- | --- | --- |
| *Nicotiana tabacuum* | crtW/crtZ | *Paracoccus* sp*.* | 35S | Nuclear | 800μg/g | 3.20% | Leaf | (Ralley et al., 2004) |
| *Nicotiana tabacuum* | bkt1 | *Haematoccocus pluvialis* | slPDS | Nuclear | 83.9μg/g | 23.50% | Nectary | (Mann et al., 2000) |
| *Nicotiana tabacuum* | crtW/crtZ | *Brevundimonas* sp. SD212 | rrn | Plastid | 3.29mg/g | 57.5% | Leaf | (Hasunuma et al., 2008) |
| *Nicotiana tabacuum* | crtZ/crtW | *Brevundimonas* sp. SD212 | rrn | Plastid | 5.44mg/g | 73.7% | Leaf | (Hasunuma et al., 2008) |
| *Nicotiana tabacuum* | crtW | *Brevundimonas* sp. SD212 | rrn | Plastid | 1.88mg/g | 49.0% | Leaf | (Hasunuma et al., 2008) |
| *Nicotiana glauca* | crtO | *Synechocystis* sp. | 35S | Nuclear | 0g | 0% | Leaf | (Gerjets et al., 2007) |
| *Nicotiana glauca* | crtW | *Nostoc punctiforme* | 35S | Nuclear | 60μg/g | 22% | Flowers | (Gerjets et al., 2007) |
| *Nicotiana glauca* | crtO | *Synechocystis* sp. | 35S | Nuclear | 0g | 0% | Leaf | (Zhu et al., 2007) |
| *Nicotiana glauca* | crtW/crtZ | *Brevundimonas* sp. SD212 | 35S | Nuclear | 140μg/g | 2.3% | Leaf | (Mortimer et al., 2017) |
| *Arabidopsis thaliana* | czBKT | *Chlorella zofingiensis* | 35S | Nuclear | 270μg/g | 11.7% | Leaf | (Zhong et al., 2011) |
| *Arabidopsis thaliana* | crBKT | *Chlamydomonas reinhardtii* | 35S | Nuclear | 1.37mg/g | 37.1% | Leaf | (Zhong et al., 2011) |
| *Arabidopsis* *thaliana* (zeaxanthin mutant) | crBKT | *Chlamydomonas reinhardtii* | 35S | Nuclear | 2.07mg/g | 42.9% | Leaf | (Zhong et al., 2011) |
| *Solanum lycopersicum* | crtW/crtZ | *Brevundimonas* sp. SD212 | 35S | Nuclear | 83 μg/g | 2.5% | Fruit | (Nogueira et al., 2017) |
| *Solanum lycopersicum* | crBKT/  hpBHY | *Chlamydomonas reinhardtii/ Haematoccocus pluvialis* | 35S | Nuclear | 2.36mg/g | 38.50% | Fruit | (Huang et al., 2013) |
| *Solanum lycopersicum*  (B-mutant) | crBKT/  hpBHY | *Chlamydomonas reinhardtii/ Haematoccocus pluvialis* | 35S | Nuclear | 3.12mg/g | 59.0% | Fruit | (Huang et al., 2013) |
| *Solanum lycopersicum* | crBKT | *Chlamydomonas reinhardtii* | 35S | Nuclear | 400μg/g | 10.60% | Fruit | (Huang et al., 2013) |
| *Solanum lycopersicum*  (B-mutant) | crBKT | *Chlamydomonas reinhardtii* | 35S | Nuclear | 350μg/g | 9.20% | Fruit | (Huang et al., 2013) |
| *Daucus carota* | hpBKT/  atBHY | *Haematoccocus pluvialis/ Arabidopsis thaliana* | d35S | Nuclear | 91.6μg/g | 26.50% | Root | (Jayaraj et al., 2008) |
| *Solanum tuberosum* | crtW/crtZ | *Brevundimonas* sp. SD212 | 35S | Nuclear | 510μg/g | 4% | Tuber | (Mortimer et al., 2016) |
| *Solanum tuberosum* | crtO | *Synechocystis* sp. | 35S | Nuclear | 0.7μg/g | 1.8% | Tuber | (Gerjets and Sandmann, 2006) |
| *Solanum tuberosum* (desiree) | hpBKT | *Haematoccocus pluvialis* | slPDS | Nuclear | 0.6μg/g | 18.80% | Tuber | (Morris et al., 2006) |
| *Solanum tuberosum* (desiree) | hpBKT/crtB | *Haematoccocus pluvialis/Erwinia uredovora* | slPDS | Nuclear | 0.2μg/g | 3.84% | Tuber | (Morris et al., 2006) |
| *Solanum tuberosum* (Mayan Gold) | hpBKT | *Haematoccocus pluvialis* | slPDS | Nuclear | 13.9μg/g | 45.70% | Tuber | (Morris et al., 2006) |
| *Oryza sativa* | crBKT/  hpBHY | *Chlamydomonas reinhardtii/ Haematoccocus pluvialis* | osGluB4 | Nuclear | 16.23μg/g | 73.97% | Endosperm | (Zhu et al., 2018) |
| *Glycine max* | crtW | *Brevundimonas* sp. SD212 | stUbi-3 | Nuclear | 7μg/g | 1.01% | Seed | (Pierce et al., 2015) |
| *Zea maize* | crBKT/crtZ | *Chlamydomonas reinhardtii/ Brevundimonas* sp. SD212 | zmγ-zein | Nuclear | 16.77μg/g | 59.62% | Endosperm | (Farré et al., 2016) |
| *Lotus japonicus* | crtW | *Agrobacterium aurantiacum* | 35S | Nuclear | 89.9μg/g | 23.2% | Flower | (Suzuki et al., 2007) |
| *Lactuca sativa* | crtW/crtZ | *Brevundimonas* sp. SD212 | rrn | Plastid | 178.02μg/g | 77.4% | Leaf | (Harada et al., 2014) |

Zeaxanthin mutant is an Arabidopsis line that accumulates zeaxanthin as the predominant carotenoid. B-mutant tomatoes are lines that accumulate high levels of β-carotene in the fruit. crtB = bacterial carotenogenic B gene encoding a phytoene synthase, crtO = bacterial carotenoid O gene encoding an alternative β-carotene ketolase, crtW = bacterial carotenogenic W gene encoding a β-carotene ketolase, crtZ = bacterial carotenogenic Z gene encoding a β-carotene hydroxylase, hpBKT = algal β-carotene ketolase from *Haematococcus pluvialis*, czBKT = algal β-carotene ketolase from *Chlorella zofingiensis*, crBKT = truncated algal β-carotene ketolase from *Chlamydomonas reinhardtii*. hpBHY = algal β-carotene hydroxylase from *Haematococcus pluvialis*. atBHY = β-carotene hydroxylase from *Arabidopsis thaliana*. 35S = CaMV 35S constitutive promoter, slPDS = Promoter of phytoene desaturase from *Solanum lycopersicum*, rrn = Plastid rRNA operon, d35S = double CaMV 35S constitutive promoter, osGluB4 = *Oryza sativa* glutelin gene promoter, stUbi-3 = *Solanum tuberosum* ubiquitin gene promoter, zmγ-zein = *Zea maize* γ-zein endosperm gene.

**Supplementary Table 5.** Oligonucleotides used for cloning, sequencing, or PCR amplifications.

| Name | Sequence | Length (bp) | Description |
| --- | --- | --- | --- |
| QA1-HBFD_F | taaataaccATGGCTCCTGTTCTCCTTGGATTGAAAC | 37 | Additional NcoI recognition site at 5’ end of HBFD1 |
| QA2-HBFD_R | atatatgcggccgcCTACTGCTTCATAATGAAGTTAATCGCTC | 43 | Additional NotI recognition site at 3’ end of HBFD1 |
| QA3-CBFD_F | taattagggcccaccATGGCAGCAGCAATTTCAGTGTTCAGTT | 43 | Additional ApaI recognition site at 5’ of CBFD2 |
| QA4-CBFD_R | atatatctcgagTCAGGTAGATGGTTGCGTTCGTTTAGTT | 40 | Additional XhoI recognition site at 3’ of CBFD2 |
| QA5-Bidir.prom.F | taattaGAGCTCGGTACCCACTGGATTTTGG | 31 | Binds to 35S terminator from 3’ end |
| QA6-Bidir.prom.R | atatatcctaggCCCGATCTAGTAACATAGATGACACCG | 39 | Additional compatible AvrII recognition site at 3’ of FMVm |
| QA7-FMVmRv | CACTATCTTCACAATAAAGTGACAGATAGC | 30 | Internal primer for Sanger sequencing HBFD1 |
| QA8-FMVmFw | TTCGCAAGACCCTTCCTCTATATAAGGA | 28 | Internal primer for Sanger sequencing CBFD2 |
| QA9-KetolaseFw | GGGCCCACCATGGCTTCCTCAATG | 24 | Internal primer in transit peptide sequence for Sanger sequencing |
| QA10-KetolaseRv | AAGCTTTCAAGACTCGCCGCGCCAC | 25 | Internal reverse primer in crtW for Sanger sequencing |
| QA11-KetocoRv | AAGCTTTCAAGACTCACCGCGCCAAAG | 27 | Internal reverse primer in Cit/crtW for Sanger sequencing |
| QA12-CBFD | CGTTAATAATAGCAAACACATCATTGAACTC | 31 | Internal primer in CBFD2 for Sanger sequencing |
| QA53-CBFDfw | ATGGCAGCAGCAATTTCAGT | 20 | Used to verify CBFD2 transgene through conventional PCR |
| QA54-CBFDrv | GGAACAGGCCAAATGGTACA | 20 | Used to verify CBFD2 transgene through conventional PCR |
| QA55-HBFDfw | TTCAACAAGCGGAGAAGTGC | 20 | Used to verify HBFD1 transgene through conventional PCR |
| QA56-HBFDrv | CAAACTCCAGGCTGCGTACT | 20 | Used to verify HBFD1 transgene through conventional PCR |
| QA57-Ketolasefw | CCTCCAGTCTGGTGATCGTC | 20 | Used to verify crtW transgene through conventional PCR |
| QA58-Ketolaserv | AAGTGGAAGCAGGTGAGCAG | 20 | Used to verify crtW transgene through conventional PCR |
| QA59-Coketofw | TGCTGGTATGATCGTTGCTG | 20 | Used to verify Cit/crtW transgene through conventional PCR |
| QA60-Coketorv | CAGTATGGCGATGAGGAAGC | 20 | Used to verify Cit/crtW transgene through conventional PCR |

Uppercases indicate bases identical to the target sequence. Lowercases indicate non-homologous restriction sites and additional bases.
